# Development and Validation of the Food Expectation Questionnaire (FEX‐Q): An Image‐Based Instrument to Assess Food‐Related Perceptions and Symptom Expectations

**DOI:** 10.1111/nmo.70464

**Published:** 2026-10-12

**Authors:** Ryo Katsumata, Stine Störsrud, Magnus Simrén, Sanna Nybacka

**Affiliations:** ^1^ Department of Molecular and Clinical Medicine Institute of Medicine, University of Gothenburg Gothenburg Sweden; ^2^ Department of Medicine Sahlgrenska University Hospital Gothenburg Sweden; ^3^ Center for Functional GI and Motility Disorders University of North Carolina/Chapel Hill Chapel Hill North Carolina USA; ^4^ Department of Life Sciences Chalmers University of Technology Gothenburg Sweden

## Abstract

**Background:**

Food‐related gastrointestinal (GI) symptoms are highly prevalent in patients with IBS. Although dietary components may trigger symptoms through luminal mechanisms, cognitive expectations may also shape symptom perception within the gut‐brain axis. No validated instrument exists to measure food‐related symptom expectations. We developed and validated the Food Expectation Questionnaire (FEX‐Q).

**Methods:**

The FEX‐Q was developed using a stepwise process including patient input, face‐to‐face validation, and an online validation. The finalized questionnaire presents food images rated on six visual analogue scale items, with food‐related GI symptom expectation as the core measure, alongside taste preference, willingness to eat, perceived healthiness, and perceived fat and carbohydrate content. Adults with IBS and non‐IBS controls completed the FEX‐Q together with validated questionnaires assessing GI symptoms, psychological distress, food‐related quality of life, and food avoidance.

**Results:**

Face‐to‐face validation supported food recognition, item comprehension, and response processes, resulting in the final 44‐image version. In the online survey including 134 patients with IBS and 126 non‐IBS controls, the FEX‐Q demonstrated known‐groups validity with higher symptom expectation scores in IBS than in non‐IBS controls (50.1 vs. 18.4). Validity of the nutrient‐perception items was supported by correlations between perceived and calculated fat (*r* = 0.78) and carbohydrate content (*r* = 0.59). Convergent validity was demonstrated through significant associations with GI symptom severity (*r* = 0.42) and food‐related quality of life (*r* = −0.39). Split‐half reliability was high (*r* = 0.88).

**Conclusion:**

The FEX‐Q is a promising instrument for assessing food‐related symptom expectations and perceptions. It may facilitate future mechanistic research into food‐related symptom generation and inform personalized dietary management in IBS.

## Key Points

- The Food Expectation Questionnaire (FEX‐Q) is a novel image‐based instrument that assesses six dimensions across 44 food images, including gastrointestinal (GI) symptom expectations, willingness to eat, and perceived nutritional characteristics.
- The FEX‐Q demonstrated good feasibility and validity and may facilitate research on food‐related GI symptom expectations and perceptions.
- Patients with IBS reported greater food‐related GI symptom expectations than non‐IBS controls, which were associated with more severe GI and psychological symptoms and impaired food‐related quality of life.

## Graphical abstract

The Food Expectation Questionnaire (FEX‐Q) is the first validated instrument designed to assess food‐related symptom expectations in IBS. Patients with IBS reported substantially higher symptom expectations than controls, and these expectations were associated with gastrointestinal symptom severity and food‐related quality of life.

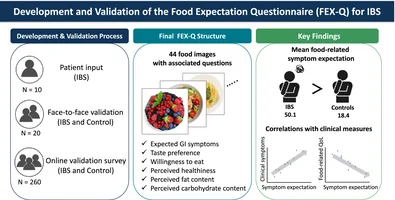

## 1 Introduction

Food‐related gastrointestinal (GI) symptoms are highly prevalent among individuals with irritable bowel syndrome (IBS) [1], one of the most common disorders of gut–brain interaction (DGBI) affecting approximately 4%–9% of the global population [2, 3]. Many patients report symptom provocation after consuming specific foods, particularly items rich in fermentable carbohydrates such as legumes, onions, wheat products, or certain fruits [1]. For this reason, dietary modifications remain a central therapeutic strategy. The low fermentable oligo‐, di‐, monosaccharide and polyol (FODMAP) diet, the most evidence‐based dietary treatment for IBS [4], likely reduces symptoms by lowering the intestinal load of fermentable substrates, thereby reducing gas production and osmotic effects [5]. However, mechanistic insights linking food exposure to symptoms are incomplete, and reduced fermentation alone cannot fully explain the variability in symptom responses.

IBS is characterized by dysregulation of the bidirectional gut–brain axis, where cognitive and affective processes shape how visceral signals are interpreted. Psychological factors, including anxiety, stress, and prior negative food experiences, can amplify symptom perception even in the absence of physiological provocation [1, 6]. Despite this, few studies have systematically examined how individuals with IBS perceive foods, how severe symptoms they expect from different foods, or how these expectations relate to actual symptom experiences. Emerging neuroimaging data suggest that simply seeing food images can activate brain regions implicated in threat processing and interoception among individuals with DGBI [7], highlighting the importance of understanding visual, cognitive, and emotional responses to food.

So far, no validated questionnaire exists to evaluate food‐related symptom expectations, perceived nutritional characteristics, and emotional or sensory food responses in IBS. To address this gap, we developed the Food Expectation Questionnaire (FEX‐Q), a tool using food images to assess taste preference, emotional responses, perceived healthiness, macronutrient estimations, and expected severity of GI symptoms after consuming each food displayed. This paper describes the development process and validation results of the FEX‐Q.

## 2 Methods

### 2.1 Study Overview

The aim of this study was to develop a visual digital questionnaire capable of reliably assessing perceptions of foods and symptom expectations in individuals with and without IBS, the FEX‐Q. This study is part of the larger project *Good Gut Feelings—Food Expectations in the Brain–Gut Axis*, conducted at Sahlgrenska University Hospital, Gothenburg, Sweden. The FEX‐Q was developed aiming for use in several ongoing and future studies, including large‐scale cross‐sectional investigations and neuroimaging studies.

The development and validation of the FEX‐Q followed a multi‐step process consisting of three sequential phases: (1) questionnaire development, including concept generation, item development, and patient input; (2) face‐to‐face validation; and (3) online quantitative validation. The study was approved by the Swedish Ethical Review Authority (Dnr 2025–02417‐01). All participants received written information about the study and provided informed consent prior to participation.

### 2.2 Questionnaire Development

The aim of the questionnaire development phase was to generate the initial FEX‐Q domains, questionnaire items, and food image set. The questionnaire development followed a multi‐step process including concept generation, item development, and patient input.

### 2.3 Concept Generation

To develop the FEX‐Q, the multidisciplinary study team consisting of dietitians (SN and SS) and gastroenterologists (RK and MS) first defined the key domains considered relevant to food‐related symptom expectations in IBS, identified via a narrative review of key publications on food‐related symptom triggers and dietary management, combined with the team's clinical experience [1, 7, 8, 9, 10, 11].

The overall structure of the FEX‐Q is illustrated in Figure 1. Briefly, participants are presented with a series of food images and rate each image across six dimensions using 0–100 visual analogue scales (VASs). Initial candidate domains included (1) taste preference, (2) expected GI symptom severity, (3) emotional response, (4) perceived healthiness, and perceived (5) fat and (6) carbohydrate content. A parallel version for healthcare professionals included questionnaire items 4, 5, and 6, and replaced the symptom expectation item 2 to address whether they believe that patients with IBS would have symptoms after consumption of the foods displayed.

**FIGURE 1:**
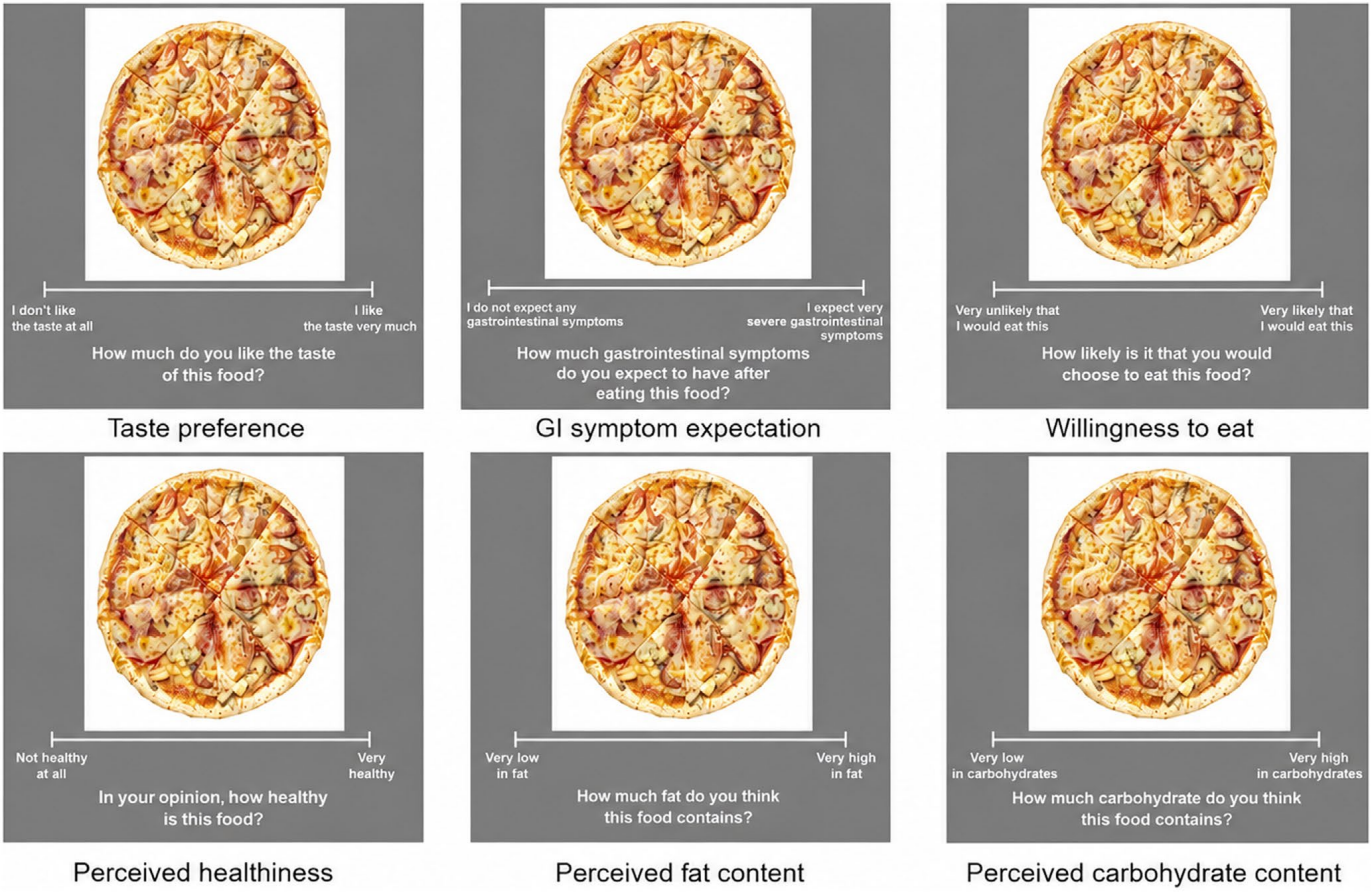
Overview of the Food Expectation Questionnaire (FEX‐Q). Participants are presented with a series of food images and rate each image across six dimensions using 0–100 visual analogue scales (VASs): taste preference, expected gastrointestinal symptom severity, willingness to eat, perceived healthiness, perceived fat content, and perceived carbohydrate content.

### 2.4 Selection of Food Images

An initial set of 48 food images was assembled based on our narrative review [1, 7, 8, 9, 10, 11] and clinical experience of our multidisciplinary team. The images were selected to represent a broad spectrum of foods, including common “trigger foods” (e.g., onion, legumes, coffee) [1, 4], perceived “safe foods” (e.g., rice, berries, bananas), foods varying in fat, carbohydrate, fiber, and FODMAP content, and foods commonly consumed in Sweden. Common foods were represented both by traditional Swedish foods (meatballs, shrimp sandwich) but also popular international foods (sushi, Thai curry, tacos, Indian thali).

### 2.5 Patient Input on Food Image Selection

To ensure that the food image set was recognizable and relevant to individuals with IBS, ten patients with IBS were invited to provide feedback individually on the initial selection of food images at the specialized neurogastroenterology outpatient clinic at Sahlgrenska University Hospital, Gothenburg, Sweden. Participants reviewed printed color images of the candidate foods and were asked whether they could identify the foods shown, whether they considered the selected foods relevant in the context of IBS, and whether any commonly perceived symptom‐triggering or symptom‐safe foods were missing. Patient feedback was transcribed in real time by the researcher during each session and subsequently reviewed by the multidisciplinary development team to refine the food image set. Within the development team, dietitians contributed expertise in food selection and nutritional composition, while gastroenterologists contributed clinical expertise regarding food‐related symptom triggers in IBS. A sample of ten participants was considered appropriate for this exploratory concept development phase [12].

### 2.6 Face‐to‐Face Validation

The primary aim of the face‐to‐face validation was to assess food image recognition, item comprehension, the response processes, and the content validity of the draft FEX‐Q. Participant feedback obtained during the face‐to‐face validation was used to further revise and improve the food images and questionnaire items.

#### 2.6.1 Participants—Face‐to‐Face Validation

Twenty adult volunteers participated in the face‐to‐face validation, including six individuals with IBS (defined according to the Rome IV diagnostic criteria) and fourteen non‐IBS controls. This sample size was considered sufficient for face‐to‐face validation, consistent with methodological studies suggesting that saturation in interview‐based questionnaire testing is often achieved within approximately 12–24 participants [13, 14].

#### 2.6.2 Procedures—Face‐to‐Face Validation

Participants completed the draft FEX‐Q using a laptop or desktop monitor in the presence of a trained researcher. The structured follow‐up questions were designed to evaluate three aspects relevant to questionnaire content validity: food recognition, item comprehension, and response reasoning. During completion, participants were asked structured follow‐up questions. Further details regarding the three key dimensions are provided below:

1. Food recognition: participants were asked to identify the food item represented in each image. “Which food item or dish do you identify in this image?”
2. Interpretation of the questions: participants were asked how they interpreted specific items, including the meaning of the term ‘gastrointestinal symptoms’. “How do you interpret this question?” and “What does ‘gastrointestinal symptoms’ mean to you in this context?”
3. Symptom rationale: participants were asked what aspects of the food image they believed might provoke symptoms. “What in this food image do you expect could provoke symptoms?”

#### 2.6.3 Evaluation Criteria—Face‐to‐Face Validation

Each item was evaluated regarding clarity of the food image, correct identification of the food, comprehensibility of the question, logical relationship between the food and perceived symptom triggers, and redundancy or overlap between items.

Responses were transcribed verbatim in real time by the researcher. Items that were repeatedly misunderstood, difficult to identify, or consistently perceived as ambiguous were flagged and those with recognition rates below 70% during the validation process were replaced with clearer alternatives or removed from the image set.

To allow assessment of response consistency and attentiveness, one duplicate image pair was intentionally included in the questionnaire. The duplicate items were presented at different positions within the questionnaire.

### 2.7 Online Survey: Quantitative Validation

The primary aim of the online validation phase was to assess the validity and reliability of the FEX‐Q in adults with IBS and non‐IBS controls. Following finalization of the questionnaire, an online survey was conducted using the FEX‐Q together with existing validated questionnaires assessing GI symptoms, psychological distress, and food‐related quality of life. Study data were collected and managed using REDCap (Research Electronic Data Capture) version 16.0.13, hosted at Sahlgrenska University Hospital [15, 16].

Participants were instructed to complete the survey using a laptop or desktop computer to ensure adequate visibility of the food images. They were also encouraged to use the “save and return” function if they experienced fatigue or reduced attention during survey completion.

#### 2.7.1 Participants—Online Survey

Adults with physician‐diagnosed irritable bowel syndrome (IBS) and non‐IBS controls were recruited by advertisement in social media platforms between October 17, 2025 and November 4, 2025. During the survey, participants were asked whether they had previously received a physician diagnosis of IBS, considered themselves to have IBS without a physician diagnosis (self‐diagnosed IBS), or did not have IBS. Only participants reporting a previous physician diagnosis of IBS were included in the IBS group. All participants were required to be able to understand Swedish in order to complete the survey. Non‐IBS controls were defined as adults without any diseases or disorders listed in the exclusion criteria. Participants were recruited nationwide across Sweden, enhancing the heterogeneity and generalizability of the validation sample.

Exclusion criteria for IBS patients included: a history of other GI diseases; severe psychiatric disorders (including weight‐ or body image–related eating disorders, such as anorexia nervosa or bulimia nervosa, or substance abuse); self‐diagnosed IBS; severe active cardiac, pulmonary, neurological, or systemic inflammatory diseases (e.g., rheumatoid arthritis); and pregnancy or breastfeeding.

Exclusion criteria for non‐IBS controls included: any history of GI disease, including IBS; severe psychiatric disorders (including weight‐ or body image–related eating disorders, such as anorexia nervosa or bulimia nervosa, or substance abuse); severe active cardiac, pulmonary, neurological, or systemic inflammatory diseases (e.g., rheumatoid arthritis); and pregnancy or breastfeeding. Participants were provided with two movie tickets as compensation for completing the survey.

#### 2.7.2 Sample Size—Online Survey

As this was a validation study, no formal a priori sample size calculation was performed. A target sample size of approximately 100 participants per group was considered sufficient to evaluate known‐groups validity, convergent validity, and reliability, and is comparable to sample sizes used in previous validation studies of patient‐reported outcome measures [17].

### 2.8 Additional Questionnaires

In addition to the FEX‐Q, participants completed a set of validated questionnaires to assess demographic characteristics, GI symptoms, psychological distress, and eating‐related behaviors:

#### 2.8.1 Demographics and Dietary Habits

Participants provided information on socio‐demographic variables, including age, sex, height, weight, education level, and occupation. Additional questions assessed general dietary habits and patterns relevant to food intake and symptom experiences.

#### 2.8.2 Gastrointestinal Symptom Rating Scale—GSRS [18] and GSRS IBS Version (GSRS‐IBS) [19]

The GSRS and GSRS‐IBS questionnaires were used to evaluate the severity of GI symptoms during the past week. In this study, the GSRS and GSRS‐IBS questionnaires were combined into a single instrument, with overlapping questions present in both questionnaires removed, resulting in a total of 19 questions (combined GSRS). These instruments assess a range of GI symptoms, including abdominal pain, bloating, diarrhea, satiety, constipation, and reflux.

#### 2.8.3 Irritable Bowel Syndrome Severity Scoring System (IBS‐SSS) [20]

The IBS‐SSS is a validated instrument used to assess IBS symptom severity over the past 10 days. It includes five domains (abdominal pain severity and frequency, bloating, bowel habit dissatisfaction, and life interference), yielding a total score ranging from 0 to 500, with higher scores indicating more severe IBS symptoms. This questionnaire was only completed by individuals with IBS.

#### 2.8.4 Hospital Anxiety and Depression Scale (HADS) [21]

The HADS is a 14‐item questionnaire designed to assess psychological distress in medical populations. It provides separate subscale scores for anxiety (HADS‐A) and depression (HADS‐D), which were calculated and used in the analyses. Higher scores indicate more severe symptoms of anxiety and depression.

#### 2.8.5 Patient Health Questionnaire‐15 (PHQ‐15) [22]

The PHQ‐15 is a 15‐item measure assessing the extent to which individuals are bothered by common somatic symptoms. It includes both GI and non‐GI symptoms and provides an overall index of somatic symptom burden, with higher scores indicating more severe symptoms.

#### 2.8.6 Food‐Related Quality of Life (FR‐QOL‐29) [23]

The FR‐QOL‐29 is a 29‐item questionnaire that evaluates quality of life specifically related to food and eating. It assesses the impact of GI symptoms on daily eating behaviors and food‐related well‐being. Higher scores indicate better food‐related QoL. This questionnaire was only completed by individuals with IBS.

#### 2.8.7 Nine‐Item Avoidant/Restrictive Food Intake Disorder Screen (NIAS) [24]

The NIAS is a 9‐item screening tool for avoidant/restrictive food intake disorder (ARFID). It consists of three subscales assessing picky eating, low appetite, and fear of aversive consequences related to eating. Higher scores indicate more symptoms associated with restrictive eating.

### 2.9 Quality Check—Online Survey

Participants with substantial missing data (> 10% of items) were excluded from the analysis. Data quality was further assessed by examining internal consistency between conceptually similar items across questionnaires. Specifically, abdominal pain–related items from the combined GSRS, IBS‐SSS, and PHQ‐15 were compared. Responses showing marked discrepancies between these measures were considered unreliable. For this purpose, responses indicating “no symptom” were defined as a score of 1 on the combined GSRS, 1 on the PHQ pain item, and < 10 on the IBS‐SSS. Conversely, responses indicating “severe symptom” were defined as scores of 6 or 7 on the combined GSRS, 3 on the PHQ, and > 90 on the IBS‐SSS. Participants who reported “no symptom” on one instrument but “severe symptom” on another were excluded due to inconsistency. Response times were also reviewed to identify questionnaires completed in an unreasonably short time. Surveys with an average completion time of less than 5 s per question were considered implausibly fast and were excluded. In addition, visual inspection of response distributions was performed, and data characterized by extreme response bias or repeated identical values across items were excluded. Finally, within‐participant response variability was examined using the standard deviation across FEX‐Q items. Extremely low variability suggestive of non‐engaged responding was considered as an additional indicator of poor data quality, which led to exclusion.

### 2.10 Outcomes—Online Survey

The primary outcome of the online survey was the mean food‐related symptom expectation score, calculated for each participant as the average symptom expectation rating across all 44 food images.

Secondary outcomes included the composite scores for the other FEX‐Q domains: taste preference, willingness to eat, perceived fat content, perceived carbohydrate content, and perceived healthiness. All outcome variables were analyzed both at the participant level and at the food image level, as appropriate.

### 2.11 Final Construct Validation

#### 2.11.1 Known‐Groups Validity: Comparison Between IBS and Control Groups

Known‐groups validity was evaluated by comparing responses between patients with IBS and non‐IBS controls. Between‐group differences in mean FEX‐Q scores were examined for each questionnaire domain.

#### 2.11.2 Construct Validity: Correspondence Between Perceived and Actual Nutrient Content

To evaluate construct validity, perceived fat and carbohydrate content ratings were compared with actual nutrient content for each food image. Actual fat and carbohydrate amounts per portion were obtained by the nutrient calculation software Dietist XP (kostdata.se, Stockholm, Sweden) and standardized recipes. These values were correlated with the mean perceived fat and carbohydrate ratings across participants using image‐level analyses.

#### 2.11.3 Convergent Validity: Associations With Established Clinical Measures

Convergent validity was assessed by examining associations between the mean food‐related symptom expectation score and established validated questionnaires. Specifically, correlations were calculated between FEX‐Q scores and measures of GI symptom severity (combined GSRS), food‐related quality of life (FR‐QoL), and avoidant/restrictive eating behaviors (NIAS).

#### 2.11.4 Reliability (Internal Consistency)

The internal consistency of the mean food‐related symptom expectation score was assessed using split‐half reliability [25]. Split‐half reliability was evaluated using 1000 random splits of the 44 items. In addition to simple random splits to make two halves, a stratified split‐half approach was performed in which items were first categorized into high (*n* = 14), middle (*n* = 16), and low (*n* = 14) scoring strata. Each half was then constructed by randomly sampling an equal number of items from each stratum.

### 2.12 Statistical Analysis

Continuous variables are presented as mean ± standard deviation (SD) or as median with interquartile range (IQR), as appropriate. Between‐group comparisons between patients with IBS and non‐IBS controls were performed using the Mann–Whitney *U* test. Associations between continuous variables were assessed using Spearman's rank correlation coefficient. Split‐half reliability was adjusted using the Spearman–Brown prophecy formula.

All statistical tests were two‐tailed, and a *p*‐value < 0.05 was considered statistically significant. All analyses were conducted using SPSS Statistics version 29.0.1.1 (IBM Corp., Armonk, NY, USA) and MATLAB R2022b (MathWorks Inc., Natick, MA, USA).

## 3 Results

### 3.1 Questionnaire Development

An initial pool of 48 food images was generated by the multidisciplinary study team. Based on patient feedback from ten patients with IBS, the image pool was revised and as a result, eight food images were replaced, and three additional food images were added to the pool, resulting in a total of 51 images for the preliminary questionnaire.

### 3.2 Face‐to‐Face Validation

#### 3.2.1 Participant Characteristics

Twenty adults participated in the face‐to‐face validation, with a mean age of 46.2 ± 16.3 years, and 65% females. Participant characteristics are summarized in Table S1.

#### 3.2.2 Food Recognition

The initial draft included 51 food images. During the face‐to‐face validation, participant feedback was used to assess image recognition, clarity, and redundancy. This iterative refinement resulted in a final set of 44 food images, all of which were correctly identified by all participants.

#### 3.2.3 Question Comprehension

All participants found the VAS questions intuitive and easy to understand. Minor wording adjustments were made to improve clarity and consistency. For example, some participants expressed uncertainty about the term “gastrointestinal symptoms.” In response, an explanatory statement was added to the first page of the questionnaire, clarifying that GI symptoms include events such as abdominal pain, gas, bloating, and heartburn.

#### 3.2.4 Symptom Expectation Reasoning

Participants based their symptom expectations on recognizable food characteristics, for example, FODMAP containing foods, fat content, portion size, and prior individual experiences. This supports the content validity of the FEX‐Q by demonstrating that responses were grounded in meaningful and clinically relevant reasoning.

#### 3.2.5 Duplicate‐Item Response Consistency

The correlation between the two presentations of the duplicate item was high (*r* = 0.77, *p* < 0.001).

#### 3.2.6 Conceptual Overlap Between Items

For the item assessing emotional response to the food images, several participants reported that their responses were primarily driven by taste preference rather than by an independent emotional reaction to the image. This interpretation was supported by a significant positive correlation between the emotional response item and taste preference ratings (*r* = 0.37, *p* = 0.007), suggesting conceptual overlap between the two constructs.

In contrast, participants reported that willingness to eat varied substantially across foods and reflected a concept distinct from taste preference. Based on this feedback, a new item was introduced: “How likely are you to eat this food?”

#### 3.2.7 Final FEX‐Q Structure

The final version of the FEX‐Q that was used in the online survey consisted of 44 food images. Representative examples of the food images are shown in Figure 2, and all 44 food images are provided in Figure S1. The structure of the final instrument, together with the VAS anchor labels and additional items evaluated during development, is summarized in Table 1. An item assessing emotional response to the food images was included in earlier development stages but was not included in the final online version due to conceptual overlap with taste preference identified during the face‐to‐face validation. The final questionnaire items used in the online survey corresponded to Q1–Q6 shown in Table 1.

**FIGURE 2:**
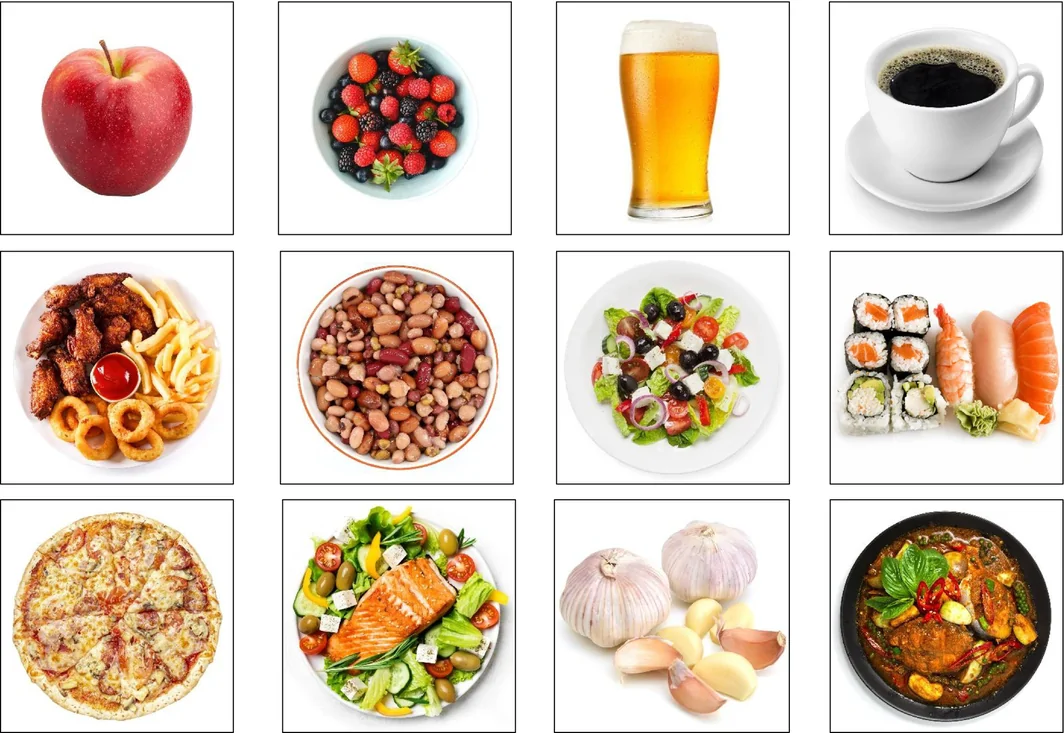
Representative examples of food images selected from the 44 food images included in the Food Expectation Questionnaire (FEX‐Q). The images represent a wide range of food categories, nutritional profiles, and foods commonly perceived to trigger gastrointestinal symptoms in patients with irritable bowel syndrome.

**TABLE 1:** Structure of the Food Expectation Questionnaire (FEX‐Q) and related items evaluated during development.

Item	Domain assessed	Example question	VAS anchors	Final version for IBS and controls	Healthcare professional version	Development outcome
Q1	Taste preference	How do you like the taste of this food?	Dislike very much—Like very much	Yes	No	Retained
Q2	Expected gastrointestinal symptom severity	How severe gastrointestinal symptoms do you expect after eating this food?	No symptoms—Severe GI symptoms	Yes	Yes (with modified wording)	Retained
Q3	Willingness to eat	How likely are you to eat this food?	Very unlikely—Very likely	Yes	No	Added after face‐to‐face validation
Q4	Perceived healthiness	How healthy do you perceive this food?	Very unhealthy—Very healthy	Yes	Yes	Retained
Q5	Perceived fat content	How much fat do you think this food contains?	Very low—Very high	Yes	Yes	Retained
Q6	Perceived carbohydrate content	How much carbohydrate do you think this food contains?	Very low—Very high	Yes	Yes	Retained
Q7	Emotional response	Which emotions does this food evoke?	Negative—Positive	No	No	Developed for future studies, but removed due to conceptual overlap with taste preference
Q8	HCP symptom expectation	How severe gastrointestinal symptoms do you believe patients with IBS would experience after eating this food?	No symptoms—Severe GI symptoms	No	Yes	Developed for future studies

Abbreviations: HCP, healthcare professionals; IBS, irritable bowel syndrome; Q1–Q8, questionnaire items 1–8.

### 3.3 Online Survey

#### 3.3.1 Participant Characteristics

A total of 332 individuals provided informed consent and initiated the online survey. Of these, 42 participants did not complete the questionnaire. Based on response time, 28 were excluded due to the short response time. In addition, two participants were excluded during data quality assessment due to marked inconsistencies between conceptually similar abdominal pain items. No additional exclusions were required based on response distribution inspection or low within‐participant variability. In total, 260 participants were included in the final analysis. This comprised 134 patients with IBS (median age 41 years, 95% female) and 126 non‐IBS controls (median age 38 years, 79% female). Demographic and clinical characteristics of each group are presented in Table 2.

**TABLE 2:** Demographic and clinical characteristics of participants from the online survey.

	Non‐IBS control	IBS	*p*
	*n* = 126	*n* = 134	
Age (years), median [IQR]	38 [28–53]	41 [31–53]	0.23
Female, *n* (%)	99 (78.6%)	127 (94.8%)	< 0.001
BMI (kg/m^2^), median [IQR]	23.3 [21.6–26.2]	24.0 [21.3–27.6]	0.74
IBS subtype, *n* (%)			
IBS‐C	—	38 (28.4)	—
IBS‐D	—	38 (28.4)	—
IBS‐M	—	38 (28.4)	—
IBS‐U	—	20 (14.9)	—
IBS‐SSS median [IQR]		305 [224.5–352.5]	

Abbreviations: BMI, body mass index; IBS, irritable bowel syndrome; IBS‐C, constipation predominant IBS; IBS‐D, diarrhea predominant IBS; IBS‐M, mixed type IBS; IBS‐SSS, irritable bowel severity scoring system; IBS‐U, unsubtyped IBS; IQR, interquartile range.

#### 3.3.2 Known‐Groups Validity

Mean scores for all FEX‐Q domains are presented in Table 3. The largest between‐group difference was observed for GI symptom expectation (Question 2 [Q2]), with patients with IBS reporting substantially higher expected symptom severity than controls (50.1 ± 12.3 vs. 18.4 ± 15.3). Patients with IBS also reported lower willingness to eat the presented foods (Q3), as well as modestly lower taste preference (Q1) and perceived healthiness (Q4). Patients with IBS also reported slightly higher perceived fat content (Q5), although the magnitude of this difference was small.

**TABLE 3:** Mean values and standard deviation for all questions to food images.

Question items mean (SD)	Non‐IBS controls	IBS	*p*
	*n* = 126	*n* = 134	
Q1. Taste preference	69.0 (9.6)	64.8 (10.1)	< 0.001
Q2. Symptom expectation	18.4 (15.3)	50.1 (12.3)	< 0.001
Q3. Willingness to eat	67.1 (11.5)	55.5 (11.9)	< 0.001
Q4. Perceived healthiness	58.3 (7.4)	56.3 (6.6)	0.01
Q5. Perceived fat content	41.1 (7.9)	43.2 (8.1)	0.02
Q6. Perceived carbohydrate content	57.8 (10.8)	57.3 (9.9)	0.34

Abbreviations: IBS, irritable bowel syndrome; Q1–Q6, questionnaire items 1–6; SD, standard deviation.

#### 3.3.3 Construct Validity: Correspondence Between Perceived and Actual Nutrient Content

At the food image level, perceived fat and carbohydrate content showed strong positive correlations with actual nutrient amounts per portion in both patients with IBS and controls (Figure 3). Perceived fat content was strongly correlated with actual fat content in both groups (IBS: *r* = 0.78, *p* < 0.001; controls: *r* = 0.78, *p* < 0.001; Figure 3A). Similarly, perceived carbohydrate content correlated significantly with actual carbohydrate content in both patients with IBS (*r* = 0.59, *p* < 0.001) and controls (*r* = 0.59, *p* < 0.001) (Figure 3B), supporting good construct validity of the FEX‐Q nutrient perception items.

**FIGURE 3:**
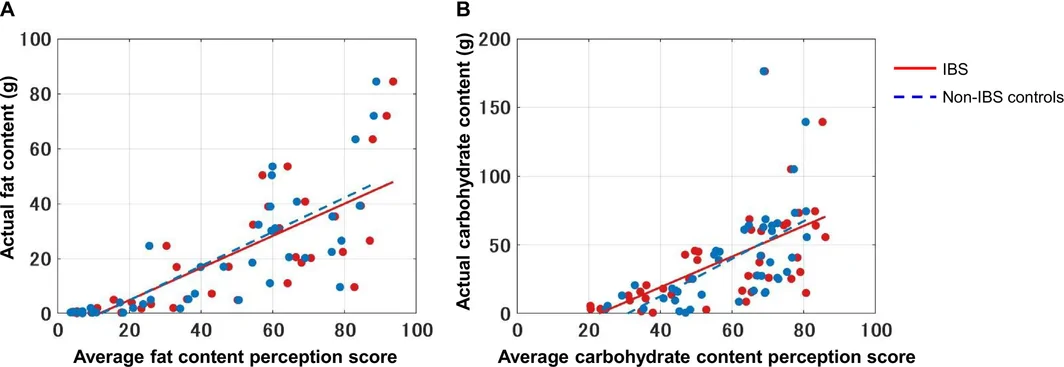
Correspondence between perceived and actual nutrient content. (A) Correlation between actual fat content and average fat perception score in both patients with IBS and non‐IBS controls. (B) Correlation between actual carbohydrate content and average carbohydrate perception score in both patients with IBS and non‐IBS controls.

#### 3.3.4 Convergent Validity: Associations With Other Variables

The mean food‐related symptom expectation score showed significant positive correlations with GI symptom severity (combined GSRS; *r* = 0.42, *p* < 0.001) and impaired food‐related quality of life (FR‐QoL; *r* = −0.39, *p* < 0.001) among patients with IBS. Moderate associations were also observed with measures of psychological distress, including HADS and PHQ scores, as well as with avoidant/restrictive eating behaviors assessed by the NIAS. The associations are illustrated in Figure 4.

**FIGURE 4:**
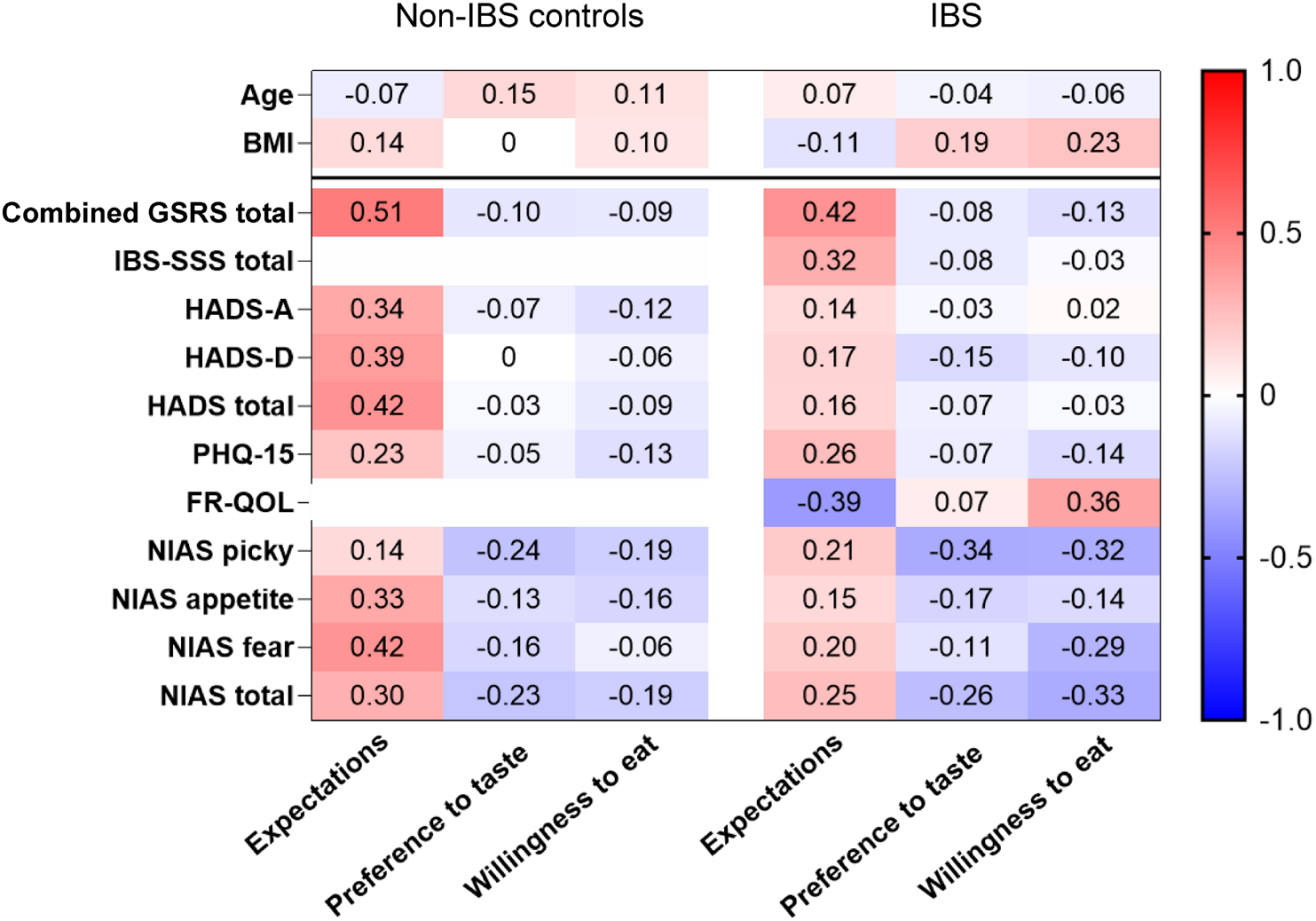
Associations between average symptom expectation scores and other clinical variables in patients with IBS and non‐IBS controls. Spearman's rank correlation coefficients (*r*) are visualized, with colors ranging from blue (*r* = −1.0) to red (*r* = 1.0). BMI, body mass index; FR‐QOL, Food‐Related Quality of Life questionnaire; GSRS, Gastrointestinal Symptom Rating Scale; HADS, Hospital Anxiety and Depression Scale; HADS‐A, Hospital Anxiety and Depression Scale–Anxiety subscale; HADS‐D, Hospital Anxiety and Depression Scale–Depression subscale; IBS, irritable bowel syndrome; IBS‐SSS, Irritable Bowel Syndrome Severity Scoring System; NIAS, Nine Item Avoidant/Restrictive Food Intake Disorder Screen; PHQ‐15, Patient Health Questionnaire‐15.

#### 3.3.5 Reliability: Internal Consistency

Split‐half reliability was calculated separately for IBS patients and non‐IBS controls. Across 1000 random splits, the median correlation between halves was *r* = 0.792 (95% CI: 0.666–0.861) in IBS patients and *r* = 0.785 (0.666–0.858) in controls. After Spearman–Brown correction (SB), reliability remained high in both groups (IBS: SB = 0.884; controls: SB = 0.880).

## 4 Discussion

In this study, the FEX‐Q was developed and validated as a novel image‐based questionnaire designed to assess food‐related perceptions and symptom expectations in individuals with IBS and in the general population. Face‐to‐face validation demonstrated that participants could accurately recognize the presented foods, understand the questionnaire items as intended, and base their symptom expectations on identifiable food characteristics, supporting strong content validity.

The present findings provide initial support for the validity and reliability of the FEX‐Q. Known‐groups validity was demonstrated by consistently higher symptom expectation scores in patients with IBS compared with controls, in line with clinical observations that individuals with IBS frequently anticipate adverse GI responses to food [1]. Construct validity was supported by strong correspondence between perceived and objectively derived nutritional characteristics of food images, indicating that responses reflected meaningful interpretations rather than arbitrary judgments. Convergent validity was further supported by significant associations between symptom expectation scores and established measures of GI symptom severity and food‐related quality of life in the expected directions. A previous study reported that IBS patients experience a lower FR‐QOL than patients with active IBD, and that IBS symptom severity is a strong predictor of impaired food‐related quality of life [23]. In addition, internal consistency was further supported by robust split‐half consistency across repeated random item partitions, which was considered appropriate given the heterogeneous image‐based structure of the FEX‐Q.

The FEX‐Q therefore provides a user‐friendly tool to investigate food‐related symptom expectations as a measurable construct. In forthcoming studies, it will be used to (1) compare expectation patterns between IBS patients and non‐IBS individuals, (2) examine psychological, dietary, and clinical correlates of these expectations, and (3) evaluate how accurately healthcare professionals anticipate patients' symptom experiences.

This study has several strengths. The FEX‐Q was developed through a rigorous, stepwise process grounded in direct patient input, ensuring strong content relevance. Face‐to‐face validation with participants confirmed clarity, interpretability, and ecological validity of the questionnaire items. A further strength is the relatively large validation sample, allowing robust quantitative evaluation of reliability and construct validity. The development process involved a multidisciplinary team, integrating expertise in gastroenterology and nutrition science. Importantly, the instrument captures not only nutritional judgments but also visual, emotional, and cognitive dimensions of food perception, providing a more comprehensive assessment than traditional diet‐based approaches.

Several limitations should be considered in this study. First, since participants involved in the concept development phase were recruited from a specialized neurogastroenterology outpatient clinic to ensure content validity, their food‐related expectations may differ from those of patients managed in primary care or general gastroenterology settings. Further, the questionnaire cannot encompass all possible foods or symptom‐relevant items. The food images included in the instrument were selected to represent culturally relevant foods, which may limit generalizability to populations with different dietary cultures. Further, the cross‐sectional design does not allow conclusions regarding causality between symptom expectations, dietary perceptions, and clinical outcomes. Additionally, replication in independent cohorts will be important to further strengthen the external validity. Future studies should establish test–retest reliability and determine the clinically meaningful interpretation of FEX‐Q scores, including thresholds for clinically important change, to support its use as an outcome measure in clinical trials.

## 5 Conclusion

The FEX‐Q is a comprehensible and clinically relevant tool for assessing food‐related perceptions and GI symptom expectations. It is now ready for application in clinical and mechanistic IBS research. Future studies will examine how different perceptual domains assessed by the FEX‐Q interact with objective dietary intake and symptom expectation.

## Author Contributions

**Ryo Katsumata:** conceptualization, methodology, formal analysis, investigation, visualization, writing – original draft. **Stine Störsrud:** conceptualization, methodology, investigation, writing – review and editing. **Magnus Simrén:** conceptualization, methodology, supervision, writing – review and editing. **Sanna Nybacka:** conceptualization, methodology, investigation, visualization, writing – review and editing.

## Conflicts of Interest

M.S. has received grants and personal fees from BioGaia; personal fees from Biocodex, Tillotts, Takeda, Sanofi, Abbvie, Janssen Immunology, Pfizer, Renapharma, Mayoly, and Bromatech. The remaining authors declare no conflicts of interest.

## Supporting information


**Table S1:** Demographic of participants from the face‐to‐face validation.

**Figure S1:** Food images included in the FEX‐Q.

## Data Availability

The data that support the findings of this study are available on request from the corresponding author. The data are not publicly available due to privacy or ethical restrictions.

## References

[1] L. Böhn , S. Störsrud , H. Törnblom , U. Bengtsson , and M. Simrén , “Self‐Reported Food‐Related Gastrointestinal Symptoms in IBS Are Common and Associated With More Severe Symptoms and Reduced Quality of Life,” American Journal of Gastroenterology 108, no. 5 (2013): 634–641. 10.1038/ajg.2013.105 23644955

[2] O. S. Palsson , B. E. Lacy , M. A. Benninga , et al., “Development of the Rome V Diagnostic Questionnaires,” Gastroenterology 170, no. 6 (2026): 1408–1416. 10.1053/j.gastro.2026.01.041 41713709

[3] A. D. Sperber , S. I. Bangdiwala , D. A. Drossman , et al., “Worldwide Prevalence and Burden of Functional Gastrointestinal Disorders, Results of Rome Foundation Global Study,” Gastroenterology 160, no. 1 (2021): 99–114.e3. 10.1053/j.gastro.2020.04.014 32294476

[4] C. J. Black , H. M. Staudacher , and A. C. Ford , “Efficacy of a Low FODMAP Diet in Irritable Bowel Syndrome: Systematic Review and Network Meta‐Analysis,” Gut 71, no. 6 (2022): 1117–1126. 10.1136/gutjnl-2021-325214 34376515

[5] D. K. Ong , S. B. Mitchell , J. S. Barrett , et al., “Manipulation of Dietary Short Chain Carbohydrates Alters the Pattern of Gas Production and Genesis of Symptoms in Irritable Bowel Syndrome,” Journal of Gastroenterology and Hepatology 25, no. 8 (2010): 1366–1373. 10.1111/j.1440-1746.2010.06370.x 20659225

[6] S. Chandran , S. N. Prakrithi , S. Mathur , M. Kishor , and T. S. S. Rao , “A Review of Functional Gastrointestinal Disorders: A Primer for Mental Health Professionals,” Archives of Mental Health 19, no. 2 (2018): 70–81.

[7] R. Katsumata , T. Hosokawa , N. Manabe , et al., “Brain Activity in Response to Food Images in Patients With Irritable Bowel Syndrome and Functional Dyspepsia,” Journal of Gastroenterology 58, no. 12 (2023): 1178–1187. 10.1007/s00535-023-02031-5 PMC10657794 37572136

[8] S. Elsenbruch , S. Ballou , L. Keefer , et al., “Biopsychosocial Aspects of Adult and Pediatric Disorders of Gut‐Brain Interaction,” Gastroenterology 170, no. 6 (2026): 1205–1223. 10.1053/j.gastro.2026.02.009 42031440

[9] W. D. Chey , J. G. Hashash , L. Manning , and L. Chang , “AGA Clinical Practice Update on the Role of Diet in Irritable Bowel Syndrome: Expert Review,” Gastroenterology 162, no. 6 (2022): 1737–1745.e5. 10.1053/j.gastro.2021.12.248 35337654

[10] Y. A. McKenzie , R. K. Bowyer , H. Leach , et al., “British Dietetic Association Systematic Review and Evidence‐Based Practice Guidelines for the Dietary Management of Irritable Bowel Syndrome in Adults (2016 Update),” Journal of Human Nutrition and Dietetics 29, no. 5 (2016): 549–575. 10.1111/jhn.12385 27272325

[11] C. Melchior , J. Algera , E. Colomier , H. Törnblom , and M. Simrén , “Irritable Bowel Syndrome With Food‐Related Symptoms: Future Directions in the Clinical Management,” United European Gastroenterology Journal 10, no. 6 (2022): 594–600. 10.1002/ueg2.12265 PMC9278572 35727703

[12] M. Hennink and B. N. Kaiser , “Sample Sizes for Saturation in Qualitative Research: A Systematic Review of Empirical Tests,” Social Science & Medicine 292 (2022): 114523. 10.1016/j.socscimed.2021.114523 34785096

[13] G. Guest , A. Bunce , and L. Johnson , “How Many Interviews Are Enough?: An Experiment With Data Saturation and Variability,” Field Methods 18, no. 1 (2006): 59–82.

[14] M. M. Hennink , B. N. Kaiser , and V. C. Marconi , “Code Saturation Versus Meaning Saturation: How Many Interviews Are Enough?,” Qualitative Health Research 27, no. 4 (2017): 591–608. 10.1177/1049732316665344 PMC9359070 27670770

[15] P. A. Harris , R. Taylor , R. Thielke , J. Payne , N. Gonzalez , and J. G. Conde , “Research Electronic Data Capture (REDCap)—A Metadata‐Driven Methodology and Workflow Process for Providing Translational Research Informatics Support,” Journal of Biomedical Informatics 42, no. 2 (2009): 377–381. 10.1016/j.jbi.2008.08.010 PMC2700030 18929686

[16] P. A. Harris , R. Taylor , B. L. Minor , et al., “The REDCap Consortium: Building an International Community of Software Platform Partners,” Journal of Biomedical Informatics 95 (2019): 103208. 10.1016/j.jbi.2019.103208 PMC7254481 31078660

[17] E. Anthoine , L. Moret , A. Regnault , V. Sébille , and J. B. Hardouin , “Sample Size Used to Validate a Scale: A Review of Publications on Newly‐Developed Patient Reported Outcomes Measures,” Health and Quality of Life Outcomes 12 (2014): 176. 10.1186/s12955-014-0176-2 PMC4275948 25492701

[18] J. Svedlund , I. Sjödin , and G. Dotevall , “GSRS—A Clinical Rating Scale for Gastrointestinal Symptoms in Patients With Irritable Bowel Syndrome and Peptic Ulcer Disease,” Digestive Diseases and Sciences 33, no. 2 (1988): 129–134. 10.1007/BF01535722 3123181

[19] I. K. Wiklund , S. Fullerton , C. J. Hawkey , et al., “An Irritable Bowel Syndrome‐Specific Symptom Questionnaire: Development and Validation,” Scandinavian Journal of Gastroenterology 38, no. 9 (2003): 947–954. 10.1080/00365520310004209 14531531

[20] C. Y. Francis , J. Morris , and P. J. Whorwell , “The Irritable Bowel Severity Scoring System: A Simple Method of Monitoring Irritable Bowel Syndrome and Its Progress,” Alimentary Pharmacology & Therapeutics 11, no. 2 (1997): 395–402. 10.1046/j.1365-2036.1997.142318000.x 9146781

[21] I. Bjelland , A. A. Dahl , T. T. Haug , and D. Neckelmann , “The Validity of the Hospital Anxiety and Depression Scale. An Updated Literature Review,” Journal of Psychosomatic Research 52, no. 2 (2002): 69–77. 10.1016/s0022-3999(01)00296-3 11832252

[22] K. Kroenke , R. L. Spitzer , and J. B. Williams , “The PHQ‐15: Validity of a New Measure for Evaluating the Severity of Somatic Symptoms,” Psychosomatic Medicine 64, no. 2 (2002): 258–266. 10.1097/00006842-200203000-00008 11914441

[23] L. Guadagnoli , E. A. Mutlu , B. Doerfler , A. Ibrahim , D. Brenner , and T. H. Taft , “Food‐Related Quality of Life in Patients With Inflammatory Bowel Disease and Irritable Bowel Syndrome,” Quality of Life Research 28, no. 8 (2019): 2195–2205. 10.1007/s11136-019-02170-4 PMC6625837 30900206

[24] H. F. Zickgraf and J. M. Ellis , “Initial Validation of the Nine Item Avoidant/Restrictive Food Intake Disorder Screen (NIAS): A Measure of Three Restrictive Eating Patterns,” Appetite 123 (2018): 32–42. 10.1016/j.appet.2017.11.111 29208483

[25] C. Spearman , “Correlation Calculated From Faulty Data,” British Journal of Psychology 3, no. 3 (1910): 271–295.

