# Supplementary material for: Development and Validation of the Food Expectation Questionnaire (FEX‐Q): An Image‐Based Instrument to Assess Food‐Related Perceptions and Symptom Expectations
Source: Neurogastroenterol Motil. 2026 Oct 12;38(10):e70464. doi: 10.1111/nmo.70464 (PMC13655752; doi:10.1111/nmo.70464)

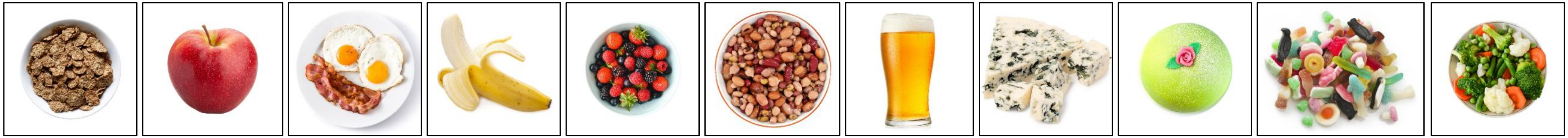

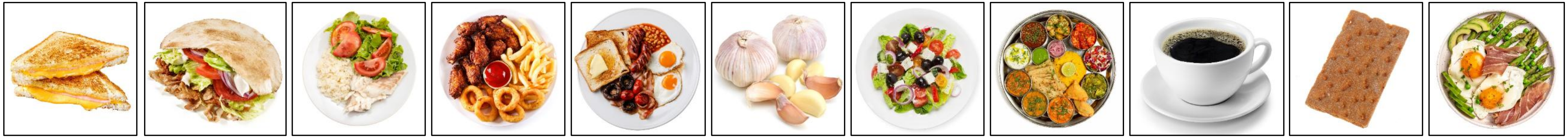

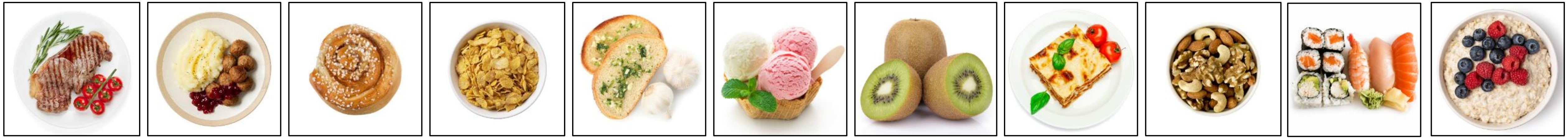

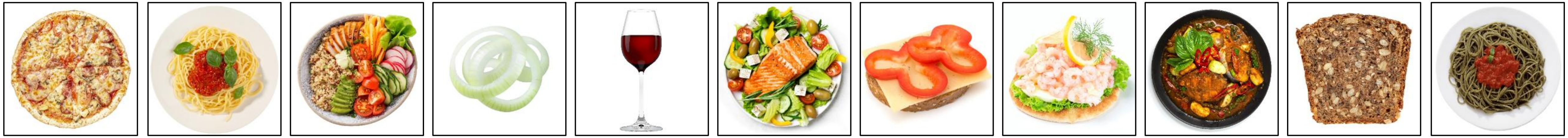

Supplement: Supplementary file 1 [file NMO-38-e70464-s001.pdf]
