# Supplementary material for: Development and Validation of the Food Expectation Questionnaire (FEX‐Q): An Image‐Based Instrument to Assess Food‐Related Perceptions and Symptom Expectations
Source: Neurogastroenterol Motil. 2026 Oct 12;38(10):e70464. doi: 10.1111/nmo.70464 (PMC13655752; doi:10.1111/nmo.70464)
Supplement: Supplementary file 2 [file NMO-38-e70464-s002.docx]

| Supplementary Table 1. Demographic of participants from the face-to-face validation. | |
| --- | --- |
|  | **Participants** n=20 |
| **Age (years), median [IQR]** | 43 [31-59] |
| **Female, n (%)** | 13 (65%) |
| **BMI (kg/m^2^), median [IQR]** | 25.1 [21.0-27.0] |
| **IBS, n (%)** | 6 (30) |

IBS, irritable bowel syndrome; BMI, body mass index; IQR, interquartile range
